# SLCO1B1 Phenotype and CYP3A5 Polymorphism Significantly Affect Atorvastatin Bioavailability

**DOI:** 10.3390/jpm11030204

**Published:** 2021-03-13

**Authors:** Pablo Zubiaur, Maria Dolores Benedicto, Gonzalo Villapalos-García, Marcos Navares-Gómez, Gina Mejía-Abril, Manuel Román, Samuel Martín-Vílchez, Dolores Ochoa, Francisco Abad-Santos

**Affiliations:** 1 Pharmacogenetics Unit, Clinical Pharmacology Department, La Princesa University Hospital Research Institute, 28006 Madrid, Spain; g.villapalos@salud.madrid.org (G.V.-G.); marcos.navares@salud.madrid.org (M.N.-G.); 2 Spanish Clinical Research Network (SCReN), La Princesa University Hospital Research Institute, 28006 Madrid, Spain; ginapaola.mejia@scren.es; 3 Universidad Autónoma de Madrid (UAM), 28029 Madrid, Spain; lolabenedicto.96@gmail.com; 4 Clinical Pharmacology Department, La Princesa University Hospital, 28006 Madrid, Spain; mdolores.ochoa@salud.madrid.org; 5 Clinical Trials Unit of La Princesa University Hospital (UECHUP), La Princesa University Hospital Research Institute, 28006 Madrid, Spain; manuel.roman@salud.madrid.org (M.R.); smvilchez@salud.madrid.org (S.M.-V.); 6 Centro de Investigación Biomédica en Red de Enfermedades Hepáticas y Digestivas (CIBERehd), ICIII, 28006 Madrid, Spain

**Keywords:** atorvastatin, pharmacogenetics, SLCO1B1, precision medicine

## Abstract

Atorvastatin, prescribed for the treatment of hypercholesterolemia, demonstrated overwhelming benefits in reducing cardiovascular morbidity and mortality. However, many patients discontinue therapy due to adverse reactions, especially myopathy. The Dutch Pharmacogenetics Working Group (DPWG) recommends an alternative agent to atorvastatin and simvastatin or a dose adjustment depending on other risk factors for statin-induced myopathy in SLCO1B1 rs4149056 CC or TC carriers. In contrast, the Clinical Pharmacogenetics Implementation Consortium (CPIC) published their guideline on simvastatin, but not on atorvastatin. In this work, we aimed to demonstrate the effect of SLCO1B1 phenotype and other variants (e.g., in *CYP3A4/5*, *UGT* enzymes or *SLC* transporters) on atorvastatin pharmacokinetics. For this purpose, a candidate-gene pharmacogenetic study was proposed. The study population comprised 156 healthy volunteers enrolled in atorvastatin bioequivalence clinical trials. The genotyping strategy comprised a total of 60 variants in 15 genes. Women showed higher exposure to atorvastatin compared to men (*p* = 0.001), however this difference disappeared after dose/weight (DW) correction. The most relevant pharmacogenetic differences were the following: AUC/DW and C_max_ /DW based on (a) SLCO1B1 phenotype (*p <* 0.001 for both) and (b) CYP3A5*3 (*p* = 0.004 and 0.018, respectively). As secondary findings: *SLC22A1* *2/*2 genotype was related to higher C_max_/DW (ANOVA *p* = 0.030) and *SLC22A1* *1/*5 genotype was associated with higher Vd/F (ANOVA *p* = 0.032) compared to *SLC22A1* *1/*1, respectively. Finally, *UGT2B7* rs7439366 *1/*1 genotype was associated with higher t_max_ as compared with the *1/*3 genotype (ANOVA *p* = 0.024). Based on our results, we suggest that SLCO1B1 is the best predictor for atorvastatin pharmacokinetic variability and that prescription should be adjusted based on it. We suggest that the CPIC should include atorvastatin in their statin-SLCO1B1 guidelines. Interesting and novel results were observed based on *CYP3A5* genotype, which should be confirmed with further studies.

Atorvastatin, prescribed for the treatment of hypercholesterolemia, demonstrated overwhelming benefits in reducing cardiovascular morbidity and mortality. However, many patients discontinue therapy due to adverse reactions, especially myopathy. The Dutch Pharmacogenetics Working Group (DPWG) recommends an alternative agent to atorvastatin and simvastatin or a dose adjustment depending on other risk factors for statin-induced myopathy in SLCO1B1 rs4149056 CC or TC carriers. In contrast, the Clinical Pharmacogenetics Implementation Consortium (CPIC) published their guideline on simvastatin, but not on atorvastatin. In this work, we aimed to demonstrate the effect of SLCO1B1 phenotype and other variants (e.g., in *CYP3A4/5*, *UGT* enzymes or *SLC* transporters) on atorvastatin pharmacokinetics. For this purpose, a candidate-gene pharmacogenetic study was proposed. The study population comprised 156 healthy volunteers enrolled in atorvastatin bioequivalence clinical trials. The genotyping strategy comprised a total of 60 variants in 15 genes. Women showed higher exposure to atorvastatin compared to men (*p* = 0.001), however this difference disappeared after dose/weight (DW) correction. The most relevant pharmacogenetic differences were the following: AUC/DW and C_max_ /DW based on (a) SLCO1B1 phenotype (*p <* 0.001 for both) and (b) CYP3A5*3 (*p* = 0.004 and 0.018, respectively). As secondary findings: *SLC22A1* *2/*2 genotype was related to higher C_max_/DW (ANOVA *p* = 0.030) and *SLC22A1* *1/*5 genotype was associated with higher Vd/F (ANOVA *p* = 0.032) compared to *SLC22A1* *1/*1, respectively. Finally, *UGT2B7* rs7439366 *1/*1 genotype was associated with higher t_max_ as compared with the *1/*3 genotype (ANOVA *p* = 0.024). Based on our results, we suggest that SLCO1B1 is the best predictor for atorvastatin pharmacokinetic variability and that prescription should be adjusted based on it. We suggest that the CPIC should include atorvastatin in their statin-SLCO1B1 guidelines. Interesting and novel results were observed based on *CYP3A5* genotype, which should be confirmed with further studies.

## 1. Introduction

Statins are the most frequently prescribed drugs for the management of hypercholesterolemia, due to their effectiveness and safety profile [1]. They all inhibit the hydroxymethylglutaryl coenzyme A (HMG-CoA) reductase, which reduces cholesterol biosynthesis and modulates lipid metabolism. Statins have an antiatherosclerotic effect correlated with the decrease in LDL cholesterol [2]. Atorvastatin is a member of the statin family with greater effectiveness in cholesterol control compared to other statins, namely lovastatin, pravastatin, simvastatin, and fluvastatin, with a similar tolerability profile [3].

Atorvastatin daily dose ranges between 10 to 80 mg, depending on initial LDL blood levels. After oral administration, atorvastatin is quickly and almost entirely absorbed (95–99%), with maximum concentrations (C_max_) reached at 1 to 2 h (t_max_) [4,5]. It suffers pronounced pre-systemic clearance at the gastrointestinal tract and first-pass hepatic clearance, which explains its low systemic bioavailability (around 12%) [5,6]. Atorvastatin binds to plasma proteins (>98%), and its volume of distribution is approximately 38 L. It undergoes cytochrome P450 (CYP) 3A4 (CYP3A4) mediated metabolism to active metabolites [5,7]. Elimination is principally biliary with apparently no significant enterohepatic recirculation. Half-life (t_1/2_) is approximately 14 h for atorvastatin and 20–30 h for its metabolites [5,7]. Atorvastatin is a substrate of the organic anion transporter polypeptides 1B1 (OATP1B1) and 1B3 (OATP1B3), encoded by *SLCO1B1* and *SLCO1B3* genes, respectively [5,8].

Genetic polymorphism is related to variability in atorvastatin pharmacokinetics, pharmacodynamics, drug exposure [9], and effectiveness [10]. However, to date, the only clinical guideline for atorvastatin dose adjustment based on a pharmacogenetic biomarker is the one published by the Dutch Pharmacogenetics Working Group (DPWG) [11]. The use of an alternative statin (e.g., fluvastatin) is recommended for patients with *SLCO1B1* rs4149056 T>C, C/C (*5/*5) or T/C (*1/*5) genotypes and additional significant risk factors for statin-induced myopathy. In other words, therapy must be adjusted in those without the *SLCO1B1* normal function (NF) phenotype (*1/*1), i.e., decreased function (DF) or poor function (PF) phenotypes (*1/*5 and *5/*5 genotypes, respectively). These recommendations are the same for simvastatin (DPWG). In addition, the Clinical Pharmacogenetics Implementation Consortium (CPIC) published their pharmacogenetic guideline for *SLCO1B1* and simvastatin [12], with similar recommendations as those from DPWG.

To confirm the influence of the SLCO1B1 phenotype on atorvastatin exposure, and the need for dose adjustments based on it, we aimed to perform a candidate gene pharmacogenetic study in healthy volunteers enrolled in bioequivalence clinical trials. In addition, we proposed to investigate the influence of single nucleotide polymorphisms (SNPs) in other genes in relation to the disposition of atorvastatin, namely *CYP3A*, other *CYP* enzymes or transporters (e.g., *ABCB1* or *SLC22A1*), as well as on atorvastatin tolerability.

## 2. Materials and Methods

### 2.1. Study Population

The study population comprised healthy volunteers enrolled in five different atorvastatin or atorvastatin/ezetimibe bioequivalence clinical trials performed at the Clinical Trial Unit of Hospital Universitario de La Princesa (UECHUP), Madrid, Spain. Study protocols were revised and approved by the Hospital’s Research Ethics Committee and by the Spanish Drugs Agency (AEMPS). Complying with Spanish and European legislation on research in humans, all of them were accomplished under the Good Clinical Practice guidelines and endorsing the Declaration of Helsinki. EUDRA-CT numbers were as follows: 2018-000082-37, 2019-002222-67, 2019-000891-41, 2019-001670-29, and 2019-000656-34. All the subjects (n = 178) provided their informed consent for their enrolment in the bioequivalence clinical trial. For the pharmacogenetic study, 156 volunteers signed a specific informed consent.

All the volunteers satisfied the inclusion criteria: being healthy males or females, aged 18 to 55. Exclusion criteria comprised the following: any organic or physical pathology, the use of any pharmacological treatment in the previous 48 h, body mass index (BMI) out of the 18 to 30 range, history of any kind of drug hypersensitivity, positive abuse drug screening, smokers, alcohol addicts or ethylic intoxication in the previous week, having donated blood within the previous month, pregnancy or breastfeeding, having participated in a similar study within the previous 3 months, grapefruit intake in the previous 48 h, swallowing difficulty, and lactose or galactose intolerance.

### 2.2. Study Design and Procedures

The current observational pharmacogenetic study was based on five independent bioequivalence clinical trials (A, B, C, D, E). In four of them (A, B, C, E), atorvastatin 80 mg film-coated formulations were used. All of them were phase I, single oral dose, open-label, crossover and randomized clinical trials; the reference formulations were Cardyl or Zarator (Pfizer, Spain). Of the latter, three were replicated (B, C, E) (i.e., with four sequences and four periods) and one was not replicated (A) (i.e., with two sequences and two periods). The fifth clinical trial (D) assessed the bioequivalence for atorvastatin/ezetimibe 80/10 mg coated bilayer tablets versus ezetimibe 10 mg tablets (Ezetrol, MSD, Madrid Spain) and atorvastatin 80 mg (Zarator, Pfizer, Spain). It was replicated, with four periods and four sequences.

In all of them, the determination of plasma concentrations was blinded. Volunteers were hospitalized from 10 h before drug intake to 12 or 24 h after dosing. Formulations were administered by oral route under fasting conditions with 240 mL of water. Blood samples were extracted in EDTA K2 tubes (a) at twenty time-points between pre-dose and 48 h after drug intake (A, B, C, E) or (b) at thirty time-points between pre-dose and 72 h after drug intake (D). Plasma was extracted by centrifugation and frozen until its shipment to an external analytical laboratory. The analytical method involved a liquid–liquid extraction procedure with tert-butyl methyl ether after which atorvastatin and an internal standard were determined by reversed phase ultra-high-performance reversed phase liquid chromatography coupled to tandem mass spectrometry (UPLC/MS/MS). Method validation satisfied the European Medicines Agency (EMA) requirements for bioequivalence demonstration.

### 2.3. Pharmacokinetic Analysis

A non-compartmental approach was used to calculate pharmacokinetic parameters. Following the trapezoidal rule, the area under the curve between pre-dose and the last time-point (t) (AUC_t_) was calculated. The terminal rate constant (k_e_) was calculated by linear regression of the log-linear part of the concentration–time curve. The AUC between t and infinite was estimated as C_t_/k_e_ (AUC_t-∞_). The AUC between 0 and ∞ was calculated as AUC_t_ + AUC_t-∞_ (AUC_∞_). Drug clearance was calculated adjusted for bioavailability (Cl/F) as dose (D) divided by AUC_∞_ and weight (W) (i.e., D/AUC*W). Similarly, the volume of distribution was calculated adjusted for bioavailability (Vd/F) as Cl/F divided by k_e_. Half-life (t_1/2_) was estimated as –ln 2/k_e_. The remaining pharmacokinetic parameters were directly obtained from the concentration–time curves: the maximum concentration (C_max_) and the time to reach the C_max_ (t_max_). The CERTARA Phoenix WinNonlin software, version 6.0 (Certara USA, Princeton, NJ, USA) was used.

### 2.4. Safety

The tolerability assessment consisted of the evaluation of abnormalities in analytical values, blood parameters, physical examination or any other clinically relevant event. Furthermore, to monitor vascular and heart function, a 12-lead electrocardiogram (ECG) was carried out at predose and 1.5 h after drug intake; in all but one study, another ECG was carried out 3–4 h after dosing. Vital signs (VS), i.e., systolic and diastolic blood pressure, heart rate and tympanic temperature, were monitored simultaneously to ECG. For the notation of adverse events (AEs), volunteers were asked for abnormalities in their health status; those reported spontaneously were additionally considered. The Spanish Pharmacovigilance System algorithm was used for causality determination [13]. Only those AEs with a definite or possible causality were considered adverse drug reactions (ADRs).

### 2.5. Genotyping, Haplotyping and Phenotyping

DNA was extracted from peripheral blood in a MagNA Pure automatic DNA extractor (Roche Applied Science, Pleasanton, CA, USA). The genotyping strategy comprised the genotyping of 60 variants in 15 genes. However, not all variants could be genotyped for all samples. Firstly, a customized genotyping array was used in an Applied Biosystems QuantStudio 12K flex qPCR instrument with an OpenArray thermal block (ThermoFisher, USA). Table 1 depicts the variants genotyped in four of the five clinical trials (n = 120). The CYP3A4*20 (rs67666821) polymorphism was genotyped by KASPar SNP Genotyping System (LGC Genomics, Herts, UK) in an ABI PRISM 7900HT Sequence Detection System (Applied Biosystems, Darmstadt, Germany. A *CYP2D6* copy number variation assay (CNV) was performed in a QuantStudio 12k flex thermal cycler with a 96-well thermal block, following the methodology previously reported [14]. The remaining samples (n = 36) could not be genotyped with the OpenArray technology. Their genotyping was outsourced to CEGEN-PRB3-ISCIII (Santiago de Compostela, Galicia, Spain), supported by grant PT17/0019, of the PE I+D+i 2013–2016, funded by ISCIII and ERDF, for the following 24 matching variants: *ABCB1* C1236T (rs1128503), C3435T (rs1045642), G2677TA (rs2032582), *ABCC2* (rs717620), CYP1A2*1B (rs2470890), *1F (rs762551), CYP2A6*9 (rs28399433), CYP2B6*5 (rs3211371), CYP2C19*17 (rs12248560) *2 (rs4244285), *3 (rs4986893), CYP2C8*2 (rs11572103), *3 (rs10509681 and rs11572080), *4 (rs1058930), CYP2C9*2 (rs1799853), *3 (rs1057910), CYP3A4*20 (rs67666821), CYP3A4*22 (rs35599367), CYP3A5*3 (rs776746), CYP3A5*6 (rs10264272), *CYP4F2* (rs2108622) and UGT1A1*28 (rs887829). Another five variants not included in the OpenArray plate were genotyped: *UGT1A1* rs35350960, rs4124874, rs4148323, *UGT2B4* rs4557343 and *UGT2B7* rs7439366.

CYP3A5 (*3, *6), *CYP2D6* (*3, *4, *5, *6, *7, *8, *9, *10, *14, *17, *41 and the gene copy number)*, CYP2C19* (*2, *3, *4, *17), *SLCO1B1* (*1B, *5), *CYP2B6* (*5 and *9) and *CYP2C9* (*2, *3) variants were used to infer the enzymatic phenotype according to CPIC guidelines [12,15,16,17,18,19]. Since not all samples were genotyped for the same variants, the absence of genotyping data was assumed to be “not mutated”. The same strategy was implemented for genotyping errors (e.g., absence of amplification). *CYP1A2* (*1C, *1F and *1B) variants were used to infer the activity score and phenotype as described in previous publications [20,21]. *SLC22A1* and *ABCB1* variants were merged into haplotypes: the absence of any variant was assigned the wild-type haplotype, the presence of one variant was assigned the heterozygous haplotype and the presence of two or more variants was assigned the mutant haplotype. Another *ABCB1* haplotype was inferred by exclusively considering C3435T, G2677T/A and C1236T variants, as these were elsewhere reported to have a greater impact on the transporter’s activity or expression levels [22].

### 2.6. Statistical Analysis

All pharmacokinetic parameters were logarithmically transformed to normalize distributions. Prior to logarithmic transformations, AUC_∞_ and C_max_ were divided by the dose/weight ratio (AUC/DW, C_max_ /DW) to correct the differences in weight between sexes or races which can produce pharmacokinetic variability. To avoid random associations, the following statistical analysis strategy was followed: initially, a univariate analysis was performed, where the mean of pharmacokinetic parameters or the incidence of adverse drug reactions (ADRs) were compared according to categorical variables (e.g., sex, race, haplotypes, phenotypes). For the comparison of means, a *t* test (variables with two categories) or an ANOVA test followed by a Bonferroni post-hoc (variables with three or more categories) were used. For the comparison of the incidence of ADRs according to categorical variables, a Chi-squared test was used. Afterwards, each pharmacokinetic parameter or ADR were individually analyzed with a multivariate analysis. Either by linear or logistic regression, pharmacokinetic parameters or ADRs were explored, respectively. As independent variables, only variables with *p <* 0.05 in the univariate analysis were explored; in addition, pharmacokinetic parameters were introduced as independent variables in the logistic regression. A Bonferroni correction for multiple comparisons was carried out; the value of *p* < 0.05 for statistical significance was divided by the number of variables introduced in the multivariate analysis. The Hardy–Weinberg equilibrium (HWE) was calculated for the genotyped variants using the *HardyWeinberg* package [23] and the R-studio v.4.0.3. software. Deviations from the equilibrium were considered Pearson’s goodness-of-fit chi-square *p* values below 0.05; other statistics (e.g., Fisher exact test) were calculated with an online software (Institute of Human Genetics, University of Munich, available at https://ihg.gsf.de/cgi-bin/hw/hwa1.pl, accessed on 5 March 2021). The remaining statistical analysis were computed in SPSS v.23.0.

## 3. Results

### 3.1. Demographic Characteristics

Study population was composed by 85 women (54%) and 71 men (46%). Men’s height, weight and body mass index (BMI) were significantly superior to that of women (*p* < 0.0001, *p* < 0.0001 and *p* = 0.005, respectively) (Table 1). Caucasian was the most prevalent race (52%) compared to Latin-Americans (45%), Black (3%) and Arabic (one male). Demographics also differed significantly according to races (Table 2). The Black or Arabic group was related to higher weight compared to Caucasians and Latin-Americans (*p* < 0.0001 and *p* = 0.005, respectively) and to higher height compared to Latin-Americans (*p* = 0.027). Moreover, Caucasians were younger than Latin-Americans and Black or Arabic volunteers (*p* < 0.0001 and *p* = 0.049, respectively) and showed lower BMI (*p* < 0.001 and *p* = 0.001). Of note, the Black or Arabic group was composed by four men and one woman.

### 3.2. Pharmacokinetics

Atorvastatin mean AUC_∞_ was 166.6 ± 89.1 ng*h/mL (183.6 ± 90.7 ng*h/mL for females and 146.3 ± 83.4 ng*h/mL for males, *p* = 0.001) and mean C_max_ was 39.0 ± 25.3 ng/mL (44.8 ± 25.5 ng/mL for females and 32.0 ± 23.3 ng/mL for males, *p <* 0.001). After DW correction, the differences disappeared (Table 3).

Eight variables were significantly related to pharmacokinetic variability in the univariate analysis; for multiple-testing correction, the level of significance in the multivariate analysis was set at *p* = 0.00625 (*p* < 0.05 divided by 8, the number of variables introduced in the multivariate analysis). Healthy volunteers enrolled in the “C” clinical trial exhibited lower AUC/DW (*p* = 0.010) and higher Cl/F (*p* = 0.007) than those enrolled in the “B” clinical trial and lower C_max_ /DW (*p* = 0.039) compared to that of volunteers enrolled in the “D” clinical trial. Moreover, Vd/F in “C” was higher than that of “B” and “C” (*p* = 0.028 and *p* = 0.037, respectively), which was confirmed in the multivariate analysis (unstandardized beta coefficient = 0.184, *p* = 0.013, model R^2^ = 0.272). The use of ezetimibe (i.e., the “D” clinical trial) was associated with higher AUC/DW compared to the other clinical trials, where ezetimibe was not administered (unstandardized beta coefficient = 0.177, *p* = 0.048, model R^2^ = 0.222), to higher C_max_/DW (ANOVA *p* = 0.029, unstandardized beta coefficient = 0.288, *p* = 0.002, model R^2^ = 0.225) and to lower Vd/F (unstandardized beta coefficient = −0.517, *p* = 0.001, model R^2^ = 0.272). Finally, Caucasians showed higher Vd/F compared to Latin-Americans (ANOVA *p* = 0.028, unstandardized beta coefficient = 0.184, *p* = 0.013, R^2^ = 0.225) (Table 3).

All variants were in Hardy–Weinberg equilibrium, except for CYP3A4*2 rs55785340, CYP3A4*6 rs4646438, *CYP2A6* rs28399433, CYP2C19*3 rs4986893, *UGT1A1* rs4124874, *UGT1A1* rs4148323, CYP2D6*4 rs3892097, CYP2D6*7 rs5030867, CYP2D6*8 rs5030865, CYP1A2*1F rs762551, CYP2B6*9 rs3745274, and CYP2B6*5 rs2279343. Eight of these SNPs showed no allelic variability (Supplementary Table S1). Genetic polymorphism was associated with atorvastatin pharmacokinetic variability. Carriers of the SLCO1B1 decreased function (DF) and poor function (PF) phenotypes were related to higher AUC/DW, C_max_/DW and to lower Vd/F and Cl/F compared to carriers of the normal function (NF) phenotype (ANOVA *p <* 0.001, *p <* 0.001, *p* = 0.002, *p <* 0.001, respectively, *p <* 0.05 after Bonferroini post-hoc) which was confirmed by multivariate analysis (unstandardized beta coefficients = 0.365, 0.332, −0.341, −0.357, *p <* 0.001, <0.001, <0.001, <0.001, and R^2^ = 0.222, 0.225, 0.272 and 0.200, respectively). Moreover, *CYP3A5* *1/*3 and *3/*3 genotypes were related to lower AUC/DW, C_max_/DW and t_max_ and to higher Cl/F compared to the *1/*1 genotype (ANOVA *p* = 0.004, 0.018, <0.001, 0.005, respectively, *p <* 0.05 after Bonferroini post-hoc); the associations for AUC/DW, C_max_/DW and Cl/F were confirmed by multivariate analysis (unstandardized beta coefficients = −0.208, −0.202, 0.189, *p=* 0.007, 0.009, 0.013 and R^2^ = 0.222, 0.225 and 0.200, respectively). Moreover, the *SLC22A1* *2/*2 genotype was related to higher C_max_/DW (ANOVA *p=* 0.030, *p* > 0.05 in the multivariate analysis) and *SLC22A1* *1/*5 genotype was associated with higher Vd/F (ANOVA *p=* 0.032, unstandardized beta coefficient= 0.535, *p* = 0.011 and R^2^ = 0.272) compared to *SLC22A1* *1/*1, respectively. Finally, *UGT2B7* rs7439366 TT genotype was associated with higher t_max_ as compared with the TC genotype (ANOVA *p* = 0.024); this variable could not be analyzed in the multivariate analysis. (Table 4).

### 3.3. Safety

No serious ADR was reported during any of the five clinical trials. No clinically relevant alteration of VS or ECG was observed. Twenty-one volunteers suffered a total of 27 ADRs. Three types of ADR were reported: first, gastrointestinal symptoms (flatulence, loose stools, or diarrhea) were reported at least once in 13 volunteers; second, headache was reported at least once in 11 volunteers; third, three cases of myalgia or arthralgia were reported at least once in three volunteers. Ten out of 13 cases (76.9%) of gastrointestinal symptoms occurred in the “E” clinical trial compared to two cases in the D clinical trial (15.4%) and one in the B clinical trial (9.8%) (*p <* 0.001). Males were related to a lower risk for developing headache (logOR = −19.054, *p <* 0.001, R^2^ (Cox and Snell) = 0.068). Pharmacokinetics or genetic polymorphism were unrelated to occurrence of ADRs.

## 4. Discussion

Statins are widely prescribed for the treatment of hypercholesterolemia, having demonstrated overwhelming benefits in reducing cardiovascular morbidity and mortality. However, a considerable percentage of patients discontinue therapy due to the occurrence of adverse reactions, mainly myopathies [24]. Therefore, the personalized prescription of these drugs is recommended to avoid excessive exposure, which may lead to ADRs. In line with the above, the DPGW published its pharmacogenetic guidelines on atorvastatin and simvastatin, where drug dose adjustment is recommended in relation to the SLCO1B1 phenotype. In contrast, the CPIC published the clinical guideline on SLCO1B1 and simvastatin but not for atorvastatin. Our interest was to demonstrate the effect of SLCO1B1 phenotype on atorvastatin pharmacokinetics, which was certainly observed and reported. Furthermore, we aimed to describe the impact of variants in other genes, demographics and the study design in atorvastatin exposure and safety.

Similar to previous works, men exhibited higher weight, height, and BMI than women [25]. Black (n = 4) and Arabic volunteers (n = 1) had to be merged in a combined group for statistical analysis. The differences related to this group in weight and height may be explained by four of the Black or Arabic volunteers being men and only one being a Black woman.

The observed mean atorvastatin pharmacokinetic parameters, e.g., AUC_∞_ = 167 ng*h/mL and C_max_ = 39 ng/mL were consistent with previous works: e.g., after a 40 mg atorvastatin dose, a mean AUC_∞_ of 96 ng*h/mL and a C_max_ of 28 ng/mL was previously reported [26]. Because 80 mg fixed-dose formulations were administered in these clinical trials, women received atorvastatin to a higher dose–weight ratio than men, which was evidenced in a significantly higher AUC_∞_ (25%) and C_max_ (40%) compared to men. These results contrast with a previous study where an 11% AUC reduction was observed in women compared to men [27]. Nevertheless, after DW correction, these differences disappeared. It could therefore be concluded that dosage strength, and not sex, is related to atorvastatin pharmacokinetic variability. Moreover, the differences observed in Vd/F between Caucasian and Black or Arabic volunteers are likely explained by the different sex distribution in both groups. Hence, again, it would be the dosage strength the responsible for the differences.

The differences observed in drug exposure according to the clinical trial design were expected due to the relatively small sample size of each clinical trial (from n = 14 to n = 39) and the different characteristics (e.g., different number of periods, sequences, reference formulations). The use of ezetimibe was related to an increased atorvastatin exposure. A possible drug–drug interaction between both drugs was interrogated previously [26]. Our results contrast with the previous consensus, in which no such interaction was demonstrated. Of note, the methodology for AUC_∞_ extrapolation in the ezetimibe clinical trial was based on AUC_0-72 h_ compared to the other clinical trials which used AUC_0-48 h_. Mean atorvastatin t_1/2_ in this study was 9.1 h. Considering five half-lives, the 95% of AUC would be covered 45.5 h after drug intake. Therefore, the sampling time (0 to 48 h vs. 0 to 72 h) will certainly not be a confounding factor. As mentioned before, the relatively small sample size of each clinical trial likely explains the observed differences. Notwithstanding, should ezetimibe increase atorvastatin exposure to the extent observed in this work (i.e., less than 10% of AUC/DW), the effects may not be relevant in the clinical setting.

As expected, SLCO1B1 phenotype was the main pharmacogenetic predictor of atorvastatin pharmacokinetic variability, which justifies a dose reduction or a drug switch in DF and PF phenotype carriers. Consequently, we suggest that the CPIC should extend their pharmacogenetic guideline on simvastatin and SLCO1B1 [12] to atorvastatin, which is congruent with DPWG recommendations [11] and with previous scientific consensus [28,29,30], and probably to other statins [28] (e.g., fluvastatin, pravastatin).

**Figure 1:**
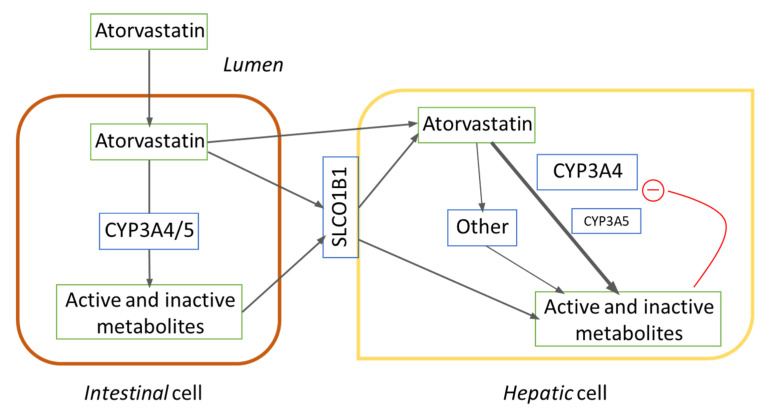
The pharmacokinetic pathway of atorvastatin focused on CYP3A and SLCO1B1.

Moreover, we identified CYP3A5*3 to be significantly related to atorvastatin pharmacokinetic variability. Our findings are controversial and require an in-depth discussion. As shown in Figure 1, CYP3A4 and CYP3A5 can metabolize atorvastatin in the intestinal and hepatic cells. Based on Ensembl data (available at: https://www.ensembl.org/index.html, accessed on 12 January 2021), the *3 allele (rs776746) has a prevalence of 80–94% in Americans and Europeans, respectively, which is consistent with our findings: approximately 87% of prevalence in a mixed population, with Caucasians, Latin-Americans mainly.

In theory, the higher metabolic capacity of *CYP3A5* expressers would be expected to result in a lower bioavailability of atorvastatin. Previous works did not observe a clinically relevant interaction [31,32]. However, these were in vitro approaches [31] or clinical observational studies with a low sample size (n = 23) [32]. In contrast, several pharmacodynamic interactions were published. *CYP3A5* *3/*3 subjects were related to higher risk of myalgia and muscle damage compared to *1/*3 subjects [33] and to increased response to atorvastatin compared to *1/*1 + *1/*3 subjects [34]. Moreover, the *3 allele was associated with increased response to statins, including atorvastatin, compared to the *1 allele [35,36]. A higher metabolism of the parent drug, assuming that its metabolites are more active, does not necessarily correlate to a lower risk for toxicity or for drug effectiveness. In contrast, CYP3A4 inhibition relates to toxicity, actually supporting that a higher metabolism indeed relates to lower effectiveness and lower risk for toxicity. Therefore, from these studies, we can conclude that the sum of atorvastatin and atorvastatin active metabolites could be higher in *CYP3A5* non-expressers, and this event relates to a higher risk for toxicity and to a better response to the drug. In our study, CYP3A5*1 (defined as the absence of *3 and *6 alleles) allele was associated with atorvastatin accumulation (e.g., carriers of *1/*1 exhibited an AUC 1.58 or 1.85 times higher than that of carriers of *1/*3 or *3/*3, respectively, *p* = 0.007). Our study is, to our knowledge, the one with the largest sample size (n = 156) published to date suggesting such an interaction. A possible explanation for this is as follows: atorvastatin, administered in the form of acid, suffers a strong first-pass metabolic effect, involving both intestinal and hepatic CYP3A4 and CYP3A5, with an oral bioavailability of 12–14% [5,6,37]. CYP3A4 is the main enzyme responsible for atorvastatin metabolism, with an intrinsic clearance 2.4 to 5 times higher than that of CYP3A5 [31]. In *CYP3A5* expressers, atorvastatin is metabolized to a wider extent in the gut, leading to active metabolites with CYP3A4 inhibitory effect. These metabolites, together with atorvastatin, reach the liver and inhibit CYP3A4, which is consistent with the previously demonstrated substrate inhibition [31]. Since in our study population a high dose of atorvastatin was administered to healthy subjects without any atorvastatin in the organism, this effect is evident. That is, the inhibitory effect is greater than the enhanced metabolic capacity. Given the high inducibility of CYP3A4, these effects are likely to be normalized over time in patients in steady state. In our study design, however, there was insufficient time for the induction of CYP3A4 expression to compensate for the inhibition associated with the CYP3A5*1 allele. Clearly, considering the controversy with these results, we encourage other researchers to proceed cautiously with them. Further studies are necessary to replicate this effect.

The *SLC22A1* gene encodes for the organic cationic transporter 1 (OCT), a transporter responsible for the hepatic uptake of xenobiotics and for the capture of organic cations from blood to epithelial cells [38]. There is very little information available regarding atorvastatin and *SLC22A1*. Atorvastatin is known to alter the in vitro expression of *SLC22A1* and in rats co-administered with nicotine [39,40]. To date, no study with a robust design or study in humans evaluated if atorvastatin is an OCT1 substrate. This is the first study to date to suggest a similar conclusion. The SLC22A1*2 allele (rs72552763) or Met420 deletion, was related to a reduced metformin uptake in vitro [41]. Here, SLC22A1*2 allele was related to a higher C_max_ compared to the *1 allele, which would be consistent with a reduced hepatic uptake and, therefore, to a reduced metabolism; this association disappeared after multivariate analysis. On the other hand, the SLC22A1*5 allele (rs34059508) was demonstrated to be another reduced-function allele and was related to a reduced metformin uptake in vitro [41]. Here, *1/*5 carriers were related to higher Vd/F. However, this association did not reach the level of significance after Bonferroni correction; these differences were probably explained by the very high standard deviation observed in the *1/*5 group, caused by the presence of outliers.

*UGT2B7*, among other UDP-glucuroniltransferases, was demonstrated to metabolize statins, including atorvastatin [42]. The *2 allele, defined by the rs7439366 variant, was previously associated with reduced activity in diclofenac and efavirenz acyl glucuronidation [43,44]. However, to the best of our knowledge, this is the first study to report an effect of this variant on atorvastatin pharmacokinetics. Here, the *1/*1 subject exhibited higher t_max_ compared to *1/*2 but not to *2/*2. Unfortunately, the number of samples analyzed for this variant (n = 36) was small and therefore these findings could be spurious.

The scarcity in ADR data is congruent with the study design, i.e., single-dose administrations. Gastrointestinal symptoms, headache and musculoskeletal ADRs are common based on atorvastatin drug label 5 which is consistent with our findings. The lower risk for headache development observed in men compared to women is likely explained by the lower exposure to atorvastatin observed in men, who were dosed to a lower dose–weight ratio.

It would be particularly interesting to validate these results in a cohort of patients chronically treated with atorvastatin for the management of hypercholesterolemia. For instance, it would be of interest to find out whether patients with a poor or reduced-function SLCO1B1 phenotype required lower doses of atorvastatin or had a higher incidence of myalgias.

### Limitations

The main limitation of this study is that the administration of a single atorvastatin dose to healthy subjects did not permit drawing any conclusion on long-term effectiveness or safety. Further studies are needed to confirm our hypotheses in a stationary state and in patients treated with atorvastatin. In contrast, bioequivalence clinical trials offer a controlled setting for evaluating pharmacokinetic variability based on genetic polymorphism or demographics as confounding factors are avoided.

## 5. Conclusions

The best predictor of atorvastatin exposure is SLCO1B1 phenotype. Accordingly, a dose adjustment could be beneficial to avoid toxicities, especially statin-related myalgias, which could lead to interruption of treatment. This conclusion is consistent with DPWG guideline on atorvastatin and SLCO1B1. We suggest that the CPIC should extend their guideline on simvastatin and SLCO1B1 to atorvastatin as the effect of the transporter phenotype on pharmacokinetics is well demonstrated. Moreover, this candidate-gene study is, to the best of our opinion, the most robust one published to date, with the highest sample size (n = 156) and the widest genotyping screening strategy. In this line, a very novel association was observed, between CYP3A5*1 and a significant increase in atorvastatin exposure. Further studies are warranted to confirm or reject our findings and hypotheses.

## Figures and Tables

**Table 1:** Variants/alleles* genotyped with the Open Array/QuantStudio 12k flex platform.

Gene	Allele/SNP	Gene	Allele/SNP
*CYP1A2*	*1C (rs2069514)	*CYP3A4*	*22 (rs35599367)
	*1F (rs762551)		rs55785340
	*1B (rs2470890)		rs4646438
*CYP2A6*	*9 (rs28399433)	*CYP3A5*	*3 (rs776746)
*CYP2B6*	*9 (rs3745274)		*6 (rs10264272)
	*5 (rs3211371)	*ABCB1*	C3435T (rs1045642)
	*4 (rs2279343)		G2677T/A (rs2032582)
	rs2279345		C1236T (rs1128503)
	rs4803419		1000-44G>T (rs10276036)
*CYP2C8*	*2 (rs11572103)		2895+3559C>T (rs7787082)
	*3 (rs10509681)		330-3208C>T (rs4728709)
	*4 (rs1058930)		2481+788T>C (rs10248420)
*CYP2C9*	*2 (rs1799853)		2686-3393T>G (rs10280101)
	*3 (rs1057910)		2320-695G>A (rs12720067)
*CYP2C19*	*2 (rs4244285)		2482-707A>G (rs11983225)
	*3 (rs4986893)		2212-372A>G (rs4148737)
	*4 (rs28399504)		rs3842
	*17 (rs12248560)	*ABCC2*	c.1247G>A (rs2273697)
*CYP2D6*	*3 (rs35742686)		rs717620
	*4 (rs3892097)	*SLCO1B1*	*1B (rs2306283)
	*6 (rs5030655)		*5 (rs4149056)
	*7 (rs5030867)		c.-910G>A (rs4149015)
	*8 (rs5030865)		rs11045879
	*9 (rs5030656)	*SLC22A1*	*2 (rs72552763)
	*10 (rs1065852)		*3 (rs12208357)
	*14 (rs5030865)		*5 (rs34059508)
	*17 (rs28371706)	*UGT1A1*	*28 (rs887829)
	*41 (rs28371725)		

* When the presence of a variant (identified with the RefSeq identifier) unequivocally defines an allele, it is indicated with the *star nomenclature.

**Table 2:** Demographic characteristics of the study population.

Sex	n	Weight (kg)	CV%	BMI (kg/m^2^)	CV%	Height (m)	CV%	Age (years)	CV%
Women	85	61.5	13.8	23.1	13.4	1.63	3.7	30.1	28.2
Men	71	75.3 *	12.7	24.4 *	10.2	1.75 *	4.0	27.8	27.0
Race									
Caucasian	81	65.5	16.8	22.6 *^2^	10.6	1.7	5.9	25.5 *^2^	22.4
Latin-American	70	69.2	15.0	24.8	11.7	1.67	6.0	32.7	26.9
Black or Arabic	5	85.3 *^1^	15.0	27.0	7.4	1.77 *^3^	5.6	33.7	18.7
Total	156	67.7	16.8	23.7	12.2	1.69	5.9	29.0	27.9

*: p < 0.05 after ANOVA or T-test compared to the other category; *^1^: *p <* 0.05 after ANOVA and Bonferroni post-hoc (Black or Arabic compared to Caucasians and Latin-Americans); *^2^*: p <* 0.05 after ANOVA and Bonferroni post-hoc (Caucasians vs. Latin-American and Black or Arabic); *^3^*: p <* 0.05 after ANOVA and Bonferroni post-hoc (Black or Arabic compared to Latin-Americans).

**Table 3:** Atorvastatin pharmacokinetic parameters based on sex, study design, use of ezetimibe, and race.

		N	AUC/DW (kg*h*ng/mL*mg)	CV%	C_max_/DW (kg*ng/mL*mg)	CV%	t_max_ (h)	CV%	t_1/2_ (h)	CV%	Vd/F (l/kg)	CV%	Cl/F (L/h*kg)	CV%
Sex	Female	85	142.6	56.7	34.3	61.2	1.4	57.1	9.3	31.2	124.0	66.3	9023.4	49.6
	Male	71	136.6	58.4	29.9	74.9	1.4	57.1	8.7	25.3	117.0	52.3	9248.5	42.7
Clinical trial	A	14	129.2	35.9	22.7	36.1	1.7	88.2	8.1	23.5	103.5	45.1	8745	35.5
	B	30	174.9	63.0	36.1	58.2	1.5	53.3	9.1	23.1	107.3	70.7	7836.7	61.5
	C	39	116.3 *^1^	59.3	26.6 *^2^	60.2	1.3	61.5	9.4	28.7	147.1 *^1^*^2^	53.0	11,036.7 *^1^	45.7
	D	37	149.4	46.3	37.4	61.0	1.4	42.9	8.7	33.3	103.9	71.9	8247.0	45.9
	E	36	130.5	57.5	33.7	82.2	1.4	42.9	9.5	30.5	127.7	52.2	9181.5	30.4
Ezetimibe	No	119	136.9	60.9	30.7	69.1	1.4	64.3	9.2	27.2	126.1	57.3	9399.1	46.2
	Yes	37	149.4 ^!^	46.3	37.4 *^!^	61.0	1.4	42.9	8.7	33.3	103.9	71.9	8247.0	45.9
Race	Caucasian	81	132.5	61.1	33.5	71.6	1.4	50.0	9.5	29.5	138.1 *^3!^	63.8	9938.9	49.4
	Latin-American	70	147.8	54.2	31.0	62.9	1.5	60.0	8.7	27.6	103.4	45.9	8279.3	38.7
	Black or Arabic	5	148.7	48.4	31.8	34.3	1.6	43.8	7.6	9.2	83.8	33.4	7808.3	36.8

SD: standard deviation; *: p < 0.05 after ANOVA or T-test compared to the other category; *^1^*: p <* 0.05 after ANOVA and Bonferroni post-hoc (C compared to B); *^2^: *p <* 0.05 after ANOVA and Bonferroni post-hoc (C compared to D); *^3^*: p <* 0.05 after ANOVA and Bonferroni post-hoc (Caucasians compared to Latin-Americans); Underlined: *p <* 0.05 after multivariate analysis (linear regression, which included the following variables: sex, study design, ezetimibe use, race, SLCO1B1 phenotype, CYP3A5*3, SLC22A1*2, and *5; *UGT2B7* rs7439366 was excluded from analysis). ^!^ *p* < 0.00625 after multivariate analysis (Bonferroni correction for multiple testing significance threshold).

**Table 4:** Atorvastatin pharmacokinetic parameters based on genotypes or phenotypes with significant variability.

		N	AUC/DW (kg*h*ng/mL*mg)	CV%	C_max_ (kg*ng/mL*mg)	CV%	t_max_ (h)	CV%	t_1/2_ (h)	CV%	Vd/F (l/kg)	CV%	Cl/F (L/h*kg)	CV%
SLCO1B1	NF	86	122.6 *^1!^	44.9	28.5 *^1!^	59.3	1.4	64.3	9	30.0	127.8 *^1!^	57.8	9827.4 *^1!^	43.1
	DF	30	181.5	59.3	37.5	60.0	1.4	57.1	8.7	25.3	99.7	78.0	7680.4	67.0
	PF	4	283.7	41.4	62.4	35.9	1.3	38.5	10.5	9.5	66.4	58.9	4382.7	60.2
CYP3A5*3	*1/*1	5	244.2 *^1^	21.5	57.1 *^1^	55.2	1.9 *^1^	42.1	9.8	26.5	65.2	41.9	4405.1 *^1^	23.7
	*1/*3	32	153.7	70.3	35.1	75.5	1.4	71.4	8.8	21.6	114.3	60.6	9181.8	55.0
	*3/*3	119	131.8	52.0	30.5	63.0	1.4	50.0	9.1	30.8	124.9	59.9	9309.2	42.7
SLC22A1*2	*1/*1	71	131.8	48.7	28.9	53.6	1.4	57.1	8.9	29.2	121.4	57.5	9393.2	47.7
	*1/*2	41	151.2	66.2	33.5	64.5	1.4	64.3	9	28.9	121.1	72.0	9136.4	54.0
	*2/*2	8	195.7	51.8	49.8 *^2^	64.7	1.6	68.8	8.9	19.1	83	59.5	6449.1	47.9
SLC22A1*5	*1/*1	114	144.6	57.0	32.3	61.9	1.4	64.3	8.9	28.1	114.8	59.7	8933.1	49.3
	*1/*5	6	105.6	58.4	24.2	34.3	1.6	43.8	10.8	30.6	193.9 *	75.6	12,455.2	56.3
*UGT2B7* rs7439366	*1/*1	9	159.6	80.6	44.1	103.9	1.8 *^3^	44.4	8.2	24.4	106.9	50.4	8821	43.8
	*1/*2	12	108.9	29.5	26.6	35.3	1.1	36.4	9.5	22.1	137.8	35.6	9990.2	22.7
	*2/*2	15	130.4	41.1	33.2	69.3	1.4	28.6	10.1	36.6	132	64.2	8751	28.3
Total		156	139.9	57.3	32.3	67.2	1.4	57.1	9.1	28.6	120.8	60.6	9125.9	46.4

NF: normal function; DF: decreased function; PF: poor function. *:p < 0.05 after ANOVA or T-test compared to the other category; *^1^*: p <* 0.05 after ANOVA and Bonferroni post-hoc (NF vs and DF and PF; *1/*1 vs. *1/*3 and *3/*3);*^2^*: p <* 0.05 after ANOVA and Bonferroni post-hoc (*2/*2 vs. *1/*1);*^3^*: p <* 0.05 after ANOVA and Bonferroni post-hoc (TT vs. TC). Underlined: *p <* 0.05 after multivariate analysis (linear regression, which included the following variables: sex, study design, ezetimibe use, race, SLCO1B1 phenotype, CYP3A5*3, SLC22A1*2, and *5; *UGT2B7* rs7439366 was excluded from analysis). ^!^ *p* < 0.00625 after multivariate analysis (Bonferroni correction for multiple testing significance threshold).

## Data Availability

The data presented in this study are available on request from the corresponding author. The data are not publicly available as they belong to the sponsors of the clinical trials.

## Supplementary Materials

The following are available online at https://www.mdpi.com/2075-4426/11/3/204/s1, Table S1.
