# Supplementary material for: SLCO1B1 Phenotype and CYP3A5 Polymorphism Significantly Affect Atorvastatin Bioavailability
Source: J Pers Med. 2021 Mar 13;11(3):204. doi: 10.3390/jpm11030204 (PMC7999651; doi:10.3390/jpm11030204)
Supplement: Supplementary file 1 [file jpm-11-00204-s001.pdf]

SNP	Tests for deviation from Hardy-Weinberg equilibrium*
ABCB1 rs4728709	n11=3 (2.27)
	n12=27 (28.46)
	n22=90 (89.27)
	p=0.573519
ABCB1 C1236T rs1128503	n11=57 (52.50)
	n12=67 (76.00)
	n22=32 (27.50)
	p=0.139243
ABCB1 C3435T rs1045642	n11=47 (47.41)
	n12=78 (77.18)
	n22=31 (31.41)
	p=0.894364
ABCB1 rs10248420	n11=10 (6.77)
	n12=37 (43.46)
	n22=73 (69.77)
	p=0.103348
ABCB1 rs10276036	n11=42 (38.53)
	n12=52 (58.93)
	n22=26 (22.53)
	p=0.197482
ABCB1 rs10280101	n11=95 (93.63)
	n12=22 (24.73)
	n22=3 (1.63)
	p=0.226049
ABCB1 rs11983225	n11=95 (93.63)
	n12=22 (24.73)
	n22=3 (1.63)
	p=0.226049
ABCB1 rs12720067	n11=97 (95.41)
	n12=20 (23.18)
	n22=3 (1.41)
	p=0.132538
ABCB1 rs2032582	n11=51 (51.92)
	n12=78 (76.15)
	n22=27 (27.92)
	p=0.762052
ABCB1 rs3842	n11=77 (76.00)
	n12=37 (39.00)
	n22=6 (5.00)

	p=0.575032
ABCB1 rs7787082	n11=10 (6.77)
	n12=37 (43.46)
	n22=73 (69.77)
	p=0.103348
ABCC2 rs2273697	n11=80 (81.67)
	n12=38 (34.65)
	n22=2 (3.67)
	p=0.289560
ABCC2 rs717620	n11=103 (105.03)
	n12=50 (45.95)
	n22=3 (5.03)
	p=0.270793
*CYP1A2**1B rs2470890	n11=22 (25.44)
	n12=82 (75.12)
	n22=52 (55.44)
	p=0.252310
*CYP1A2**1C rs2069514	n11=83 (81.67)
	n12=32 (34.65)
	n22=5 (3.67)
	p=0.402151
*CYP1A2**1F rs762551	n11=68 (75.46)
	n12=81 (66.07)
	n22=7 (14.46)
	p=0.004779
CYP2A6 rs28399433	n11=135 (132.00)
	n12=17 (23.00)
	n22=4 (1.00)
	p=0.001126
*CYP2B6**5 rs3211371	n11=134 (134.78)
	n12=22 (20.45)
	n22=0 (0.78)
	p=0.343375
*CYP2B6**9 rs3745274	n11=64 (58.80)
	n12=40 (50.40)
	n22=16 (10.80)
	p=0.023794
CYP2B6 rs2279343	n11=29 (35.21)
	n12=72 (59.58)
	n22=19 (25.21)
	p=0.022441

CYP2B6 rs2279345	n11=66 (61.95)
	n12=39 (47.10)
	n22=13 (8.95)
	p=0.061813
CYP2B6 rs4803419	n11=54 (54.67)
	n12=54 (52.65)
	n22=12 (12.68)
	p=0.778800
*CYP2C19**17 rs12248560	n11=102 (103.39)
	n12=50 (47.22)
	n22=4 (5.39)
	p=0.461790
*CYP2C19**2 rs4244285	n11=123 (122.08)
	n12=30 (31.85)
	n22=3 (2.08)
	p=0.469030
*CYP2C19**3 rs4986893	n11=156 (0.00)
	n12=0 (0.00)
	n22=0 (0.00)
	p=0.000e+00
*CYP2C19**4 rs28399504	n11=155 (155.00)
	n12=1 (1.00)
	n22=0 (0.00)
	p=0.967965
*CYP2C8**2 rs11572103	n11=148 (148.10)
	n12=8 (7.79)
	n22=0 (0.10)
	p=0.742394
*CYP2C8**3 rs10509681	n11=126 (123.85)
	n12=26 (30.29)
	n22=4 (1.85)
	p=0.076612
*CYP2C8**4 rs1058930	n11=142 (142.31)
	n12=14 (13.37)
	n22=0 (0.31)
	p=0.557353
*CYP2C9**2 rs1799853	n11=127 (124.75)
	n12=25 (29.51)
	n22=4 (1.75)
	p=0.056300
*CYP2C9**3 rs1057910	n11=143 (142.31)

	n12=12 (13.37)
	n22=1 (0.31)
	p=0.200077
*CYP2D6**10 rs1065852	n11=84 (82.80)
	n12=22 (24.40)
	n22=3 (1.80)
	p=0.303794
*CYP2D6**14 rs5030865	n11=115 (115.05)
	n12=5 (4.90)
	n22=0 (0.05)
	p=0.815704
*CYP2D6**17 rs28371706	n11=118 (118.01)
	n12=2 (1.98)
	n22=0 (0.01)
	p=0.926655
*CYP2D6**3 rs35742686	n11=118 (118.01)
	n12=2 (1.98)
	n22=0 (0.01)
	p=0.926655
CYP2D6 *4 rs3892097	n11=95 (92.44)
	n12=18 (23.11)
	n22=4 (1.44)
	p=0.016750
CYP2D6 *41 rs28371725	n11=101 (100.83)
	n12=18 (18.33)
	n22=1 (0.83)
	p=0.842128
*CYP2D6**6 rs5030655	n11=88 (88.04)
	n12=4 (3.91)
	n22=0 (0.04)
	p=0.831211
*CYP2D6**7 rs5030867	n11=120 (0.00)
	n12=0 (0.00)
	n22=0 (0.00)
	p=0.000e+00
*CYP2D6**8 rs5030865	n11=120 (0.00)
	n12=0 (0.00)
	n22=0 (0.00)
	p=0.000e+00
*CYP2D6**9 rs5030656	n11=113 (113.10)
	n12=7 (6.80)

	n22=0 (0.10)
	p=0.742077
*CYP3A4**2 rs55785340	n11=120 (0.00)
	n12=0 (0.00)
	n22=0 (0.00)
	p=0.000e+00
*CYP3A4**20	n11=153 (153.01)
	n12=3 (2.97)
	n22=0 (0.01)
	p=0.903483
*CYP3A4**22 rs35599367	n11=115 (115.05)
	n12=5 (4.90)
	n22=0 (0.05)
	p=0.815704
*CYP3A4**6 rs4646438	n11=120 (0.00)
	n12=0 (0.00)
	n22=0 (0.00)
	p=0.000e+00
*CYP3A5**3 rs776746	n11=5 (2.83)
	n12=32 (36.35)
	n22=119 (116.83)
	p=0.135303
*CYP3A5**6 rs10264272	n11=151 (151.04)
	n12=5 (4.92)
	n22=0 (0.04)
	p=0.838807
CYP4F2 rs2108622	n11=69 (70.45)
	n12=71 (68.09)
	n22=15 (16.45)
	p=0.595140
*SLC22A1**2 rs72552763	n11=71 (69.77)
	n12=41 (43.46)
	n22=8 (6.77)
	p=0.534825
*SLC22A1**3 rs12208357	n11=114 (114.08)
	n12=6 (5.85)
	n22=0 (0.07)
	p=0.778800
*SLC22A1**5 rs34059508	n11=114 (114.08)
	n12=6 (5.85)
	n22=0 (0.07)

	p=0.778800
SLCO1B1 rs11045879	n11=90 (91.00)
	n12=29 (27.00)
	n22=1 (2.00)
	p=0.416071
SLCO1B1 rs2306283	n11=22 (23.85)
	n12=63 (59.30)
	n22=35 (36.85)
	p=0.493775
SLCO1B1 rs4149015	n11=104 (104.53)
	n12=16 (14.93)
	n22=0 (0.53)
	p=0.433944
SLCO1B1 rs4149056	n11=92 (91.88)
	n12=26 (26.25)
	n22=2 (1.88)
	p=0.916909
UGT1A1 rs35350960	n11=69 (66.04)
	n12=65 (70.92)
	n22=22 (19.04)
	p=0.297146
UGT1A1 rs4124874	n11=36 (0.00)
	n12=0 (0.00)
	n22=0 (0.00)
	p=0.000e+00
UGT1A1 rs4148323	n11=36 (0.00)
	n12=0 (0.00)
	n22=0 (0.00)
	p=0.000e+00
UGT1A1 rs887829	n11=53 (52.67)
	n12=53 (53.66)
	n22=14 (13.67)
	p=0.892422
UGT2B4 rs4557343	n11=10 (11.11)
	n12=20 (17.78)
	n22=6 (7.11)
	p=0.453255
UGT2B7 rs7439366	n11=16 (13.44)
	n12=12 (17.11)
	n22=8 (5.44)
	p=0.073100

* n11: number of individuals carrying allele 1 in homozygosity; n12: number of heterozygous individuals; n22: number of individuals carrying allele 2 in homozygosity. Data in brackets represent the expected allele frequencies according to Hardy Weinberg equilibrium; *p* values correspond to the result of a Pearson's chi-squared test.
